# Round the Hospitals

**Published:** 1916-07-22

**Authors:** 


### ROUND THE HOSPITALS.

The work of construction, so far as the College of Nursing, Limited, is concerned, proceeds quietly on its way. The agreed Bill for the Registration of Trained Nurses in Great Britain has at length assumed a form which must approximate that in which it will be presented to Parliament at an autumn session, if all goes well. One of the first things the Bill proposes to do is to eliminate the word "Limited" from the title of the College, and give it statutory powers as the General Nursing Council. The Register of Trained Nurses, when it is set up, will probably prove to be a trinity of nursing—*i.e.* three registers: one for certificated trained nurses, one for male nurses, and one for mental nurses. The necessary preliminaries in preparation for action in Parliament are proceeding, we have reason to believe, with commendable smoothness, and the outlook for a settlement of the many difficulties which have troubled the profession of nursing in these islands is encouraging.

In view of this promising outlook for the College of Nursing, it behoves those matrons and sisters, and the more intelligent and far-seeing nurses, to prepare their plans with a view to enable every one of the nurses in whom they are individually interested, or for whom they may have been in any way responsible, to be made aware of the opportunity which those nurses who are most alert to their own interests will be enabled to grasp during the month of August. Once the Registration Bill is agreed to and the draft Bill settled, the Register will be open to every certificated trained nurse. Who amongst the many thousands of trained nurses will succeed in being amongst the first 7,000 to place their names on the Register, to attain the proud position of being one of the promoters of the College of Nursing, and so be recorded as worthy representatives of the best British nursing in 1916? The first 7,000 may find a place in history. For they may win the gratitude of the whole profession of trained nurses, who may come to recognise that they largely owe to them their charter of freedom, high privileges, and secured position among the working women of the Empire. Some hundreds of nurses have sent us their names, so that there is evidence already of the existence of many alert and wise virgins in the ranks. We can well imagine that the best of the matrons and the wisest will exhibit their wisdom by securing that the most worthy and capable of the nurses who have worked under them shall not miss the proud privilege of being one of the promoters of the College, which we have reason to believe will rapidly become the Royal College of Nursing.

It may be well to prevent disappointment by plainly intimating that our representative has been informed in reply to inquiries that there is no further official notice of any kind at present ready for publication in regard to the College. We have simply given our forecast, based upon experience and an intimate knowledge of the inner life and movement amongst sections of nurses and those interested in them which have given rise to discussion and led to important developments during the last forty years. Further disputes will not be business in any sense for anybody connected with nursing, for the moment is opportune to secure by agreement an approved Bill setting up registration and for the College power to secure the highest education and the highest standard for trained nurses. The latter should include the full protection of the uniform worn by every registered nurse, with the provision of further training-schools to enable the output of trained nurses to be brought to a point when it shall prove wide enough to place the schools and the profession upon an adequate basis to meet every requirement. Meanwhile all nurses should realise that the day already in the making for British nursing may arrive so promptly as to take all but the wise virgins by surprise. We could wish meanwhile that the left-handed attempts which are being made by those who ought to know better to create divisions and to manœuvre certain personalities into positions which they are not qualified to fill should, as a matter of fact, in all fairness and reason end. Such proceedings must prove abortive, and further persistence in them can only mark out the responsible initiators as unworthy of the best traditions of British nursing, for they will by persistent action demonstrate themselves to be not the friends but the enemies of certificated nurses.

Matrons will be wise to encourage the scheme for providing a short course of instruction in pathological laboratory methods which it is hoped will be arranged with a view to fitting girls who have already learned the use of the microscope, and had a grounding in inorganic chemistry, for posts as laboratory assistants. The salaries quoted for skilled laboratory assistants in military hospitals is 26s. to 30s. weekly, and the provision of suitably trained women capable of rendering intelligent assistance in the staining of slides, the making of culture media, etc., which are now done by the medical staff, would do much to expedite the work of diagnosis which is so often delayed on account of more urgent calls on the time of the pathologist. There is no doubt that capable girls who prove themselves both accurate in the scientific part of the work and practically useful in keeping bottles, test-tubes, and other appliances clean and tidy, would prove of great assistance to the over-worked medical staff, and through them to the patients.

The question of how far women workers who now replace men in the various departments of military hospitals should be under the control of the matron is one of the problems of the day. Women dispensers, clerks, and store-keepers are now to be found in many hospitals, and the control of them, and under which departmental chief they shall serve, are questions yet to be finally determined. We learn that there is a strong prejudice amongst newly engaged women workers of the class referred to against the matron's authority. Those who know the details of the working wish more could be done to convince the women workers that each has much to gain from the interest and support of a wide-minded matron, who can help her to obtain needed appliances or equipment for her work. Some matrons hold that if the nursing staff and lay helpers alike could be made to look on the matron as their representative—as one who exists to help them in difficulties, to co-ordinate their work with each other, to safeguard their health, and contribute to their comfort—they would gladly welcome supervision. It need not in any way curtail their freedom in reasonable directions, and should tend to promote a spirit of loyalty. We should welcome the views of matrons and workers on the points we have dealt with.

For Matrons' and Sisters' Appointments see page 398.

